# Intercostal Muscle Abscesses in Infective Endocarditis Associated With Migratory Deposition of Calcium Pyrophosphate

**DOI:** 10.7759/cureus.21396

**Published:** 2022-01-18

**Authors:** Yuko Nakayama, Ryuichi Ohta, Naoto Mouri, Chiaki Sano

**Affiliations:** 1 Family Medicine, Shimane University Faculty of Medicine, Izumo, JPN; 2 Community Care, Unnan City Hospital, Unnan, JPN; 3 Community Medicine Management, Shimane University Faculty of Medicine, Izumo, JPN

**Keywords:** staphylococcus aureus, intercostal muscles, bacterial, endocarditis, calcium pyrophosphate, bacteremia

## Abstract

Infective endocarditis (IE) is caused by vegetations, consisting of platelets, fibrin, inflammatory cells, and microcolonies of bacteria, fungi, rickettsia, chlamydia, and viruses, that form in the heart valves, endocardium, and large vessel intima. *Staphylococcus aureus* endocarditis is highly tissue destructive, usually follows an acute course, and tends to become severe due to valve destruction, surrounding abscesses, and distant seeding. The main complications of IE due to *S. aureus* are heart failure due to destruction of tendon cords and valves, perivalvular abscesses and fistulas, and the dissemination of septic emboli to various organs including the brain, kidney, spleen, and lungs. The most common deep tissue abscess formed is an iliopsoas abscess; however, a few publications have described the formation of superficial muscle abscesses due to *S. aureus* bacteremia. For muscles near joints, deposition of calcium pyrophosphate crystals, as seen in pseudogout, can lead to pseudo-abscess formation and increase susceptibility to infection. This has been previously recognized in the iliopsoas muscle, in particular. We report a case of IE and intercostal muscle abscesses caused by *S. aureus* bacteremia in an 86-year-old man. Careful follow-up is required in patients with IE, due to the possibility of abscess formation. Furthermore, calcium pyrophosphate deposition in muscles around joints can trigger abscess formation when there is concurrent bloodstream infection.

## Introduction

Infective endocarditis (IE) is caused by vegetations, consisting of platelets, fibrin, inflammatory cells, and microcolonies of microorganisms such as bacteria, fungi, rickettsia, chlamydia, and viruses, that form in the heart valves, endocardium, and large vessel intima [1]. IE is a systemic septic disease and has various clinical presentations, including bacteremia, vascular embolization, and heart disorders [2]. It rapidly damages the endocardium or heart valve membranes and leads to death within a few weeks if not treated [2,3].

Although many bacteria and fungi sporadically cause endocarditis, streptococci from the oral cavity and staphylococci from the skin are the predominant cause of endocarditis that occurs in native heart valves [4,5]. Staphylococci are the most common cause of IE in developed countries [2]. In particular, *Staphylococcus aureus* infection is highly tissue destructive, usually follows an acute course, and causes severe illness, due to the development of valve destruction, surrounding abscesses, and distant seedings [4]. IE due to *S. aureus* has a higher probability of stroke, systemic embolism, persistent bloodstream infection, and death than IE caused by other pathogens, and four to eight weeks of antibiotic treatment is recommended [6,7].

The main complications of *S. aureus* IE are heart failure due to destruction of the heart tendon cords and valves, perivalvular abscess and fistula formation, embolic events, and spread of infection to peripheral organs including the brain, spleen, kidney, and lung [2]. Abscess formation is commonly associated with aortic or artificial valves. IE and deep tissue abscess are reported to occur in 39% and 18%, respectively, of cases of *S. aureus* bacteremia [8]. In a retrospective cohort study of Methicillin-sensitive *S. aureus* (MSSA) bacteremia, metastatic infections occurred in 19% of patients, including IE, septic pulmonary abscess, spondylitis, lumbar abscess, epidural abscess, and septic arthritis [9].

The most common deep tissue abscess is an iliopsoas abscess. In one study, the causative bacteria were *S. aureus* (MSSA, 27%) and *Escherichia coli* (18%) [2]. There are a few reports on the formation of superficial muscle abscesses due to *S. aureus* bacteremia [10,11]; however, there are no reports of intercostal muscle abscess formation as a result of metastatic infection. Rather, most intercostal muscle abscesses are due to direct infiltration from tuberculosis or lung abscesses [10,11].

Furthermore, the pseudo-abscess formation can occur in muscles near joints. Susceptibility to infection may be enhanced by the deposition of calcium pyrophosphate. This occurs most frequently in the iliopsoas muscle [12,13]. To our knowledge, there have been no previous reports of abscess formation in the intercostal muscles as a complication of IE. We report a case of IE and intercostal muscle abscess caused by *S. aureus* bacteremia in an older man. The unique characteristics of this case highlight the need to raise awareness of the possibility of intercostal muscle abscess formation.

## Case presentation

An 86-year-old man, who lived with his eldest son, was independent in activities of daily living. Seven weeks before presentation, he was hospitalized elsewhere with a burn on the medial condyle of his right foot, caused by hot water. He developed a bruise on his left anterior chest wall three days before presentation. On the day of admission, he experienced left anterior chest pain, which worsened with coughing, and he had difficulty raising his left hand. His medical history was unremarkable. He was not taking any medications.

Initial vital signs were a body temperature of 37.7°C, blood pressure of 150/67 mmHg, the pulse of 72 beats/min, respiratory rate of 20 times/min, and saturation of percutaneous oxygen (SpO_2_) of 98%. Physical findings showed no obvious erosions in the oral cavity, no enlargement of the cervical lymph nodes, and no cardiac murmurs. There was mild redness extending from the left side of the neck to the chest wall and redness and tenderness extending from the left clavicle to the fifth intercostal space. The patient had a burn scar on the medial condyle of his right foot, but there was no apparent redness or tenderness. The initial laboratory data were shown in Table 1.

**Table 1:** Initial laboratory data SARS-CoV-2: severe acute respiratory syndrome coronavirus 2.

Marker	Level	Reference range
White blood cells	13.6	3.5–9.1 × 10^3^/μL
Neutrophils	86.9	44.0%–72.0%
Lymphocytes	5.2	18.0%–59.0%
Monocytes	7.4	0.0%–12.0%
Eosinophils	0.4	0.0%–10.0%
Basophils	0.1	0.0%–3.0%
Red blood cells	4.41	3.76–5.50 × 10^6^/μL
Hemoglobin	12.3	11.3–15.2 g/dL
Hematocrit	38.8	33.4%–44.9%
Mean corpuscular volume	88.0	79.0–100.0 fL
Platelets	16.9	13.0–36.9 × 10^4^/μL
Total protein	7.4	6.5–8.3 g/dL
Albumin	3.8	3.8–5.3 g/dL
Total bilirubin	1.7	0.2–1.2 mg/dL
Aspartate aminotransferase	31	8–38 IU/L
Alanine aminotransferase	33	4–43 IU/L
Lactate dehydrogenase	296	121–245 U/L
Blood urea nitrogen	14.8	8–20 mg/dL
Creatinine	0.76	0.40–1.10 mg/dL
Estimated glomerular filtration rate	72.9	>60.0 mL/min/L
Serum Na^+^	136	135–150 mEq/L
Serum K^+^	3.2	3.5–5.3 mEq/L
Serum Cl^-^	97	98–110 mEq/L
Creatinine kinase	238	56–244 U/L
C-reactive protein	16.3	<0.30 mg/dL
SARS-CoV-2 antigen	Negative	
Urine test		
Leukocyte	Negative	
Nitrite	Negative	
Protein	Negative	
Glucose	Negative	
Urobilinogen	Negative	
Bilirubin	Negative	
Ketone	Negative	
Blood	Negative	
pH	7.0	
Specific gravity	1.007	
Fecal occult blood	Negative	

**Figure 1:**
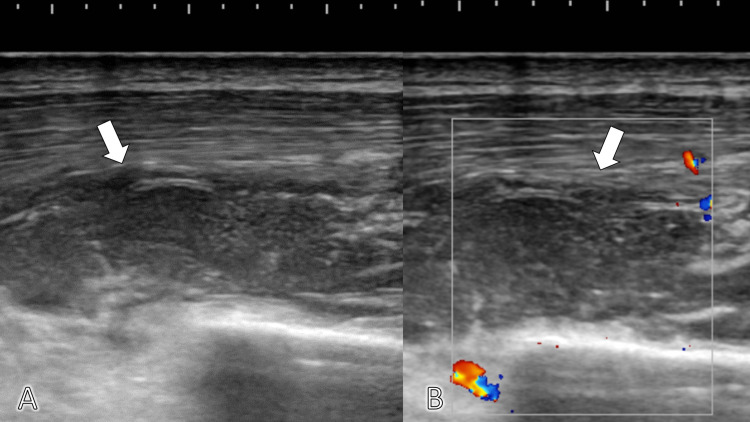
Soft-tissue ultrasonography Soft-tissue ultrasonography of the left anterior chest wall showing (A) fluid retention in the left intercostal muscle (white arrow) and (B) the increase in vascular flow (white arrow; color spotting).

We performed soft-tissue ultrasonography of the left anterior chest and found low space in the left intercostal muscle and pectoralis minor muscle and fine flow around it (Figures 1A, 1B).

**Figure 2:**
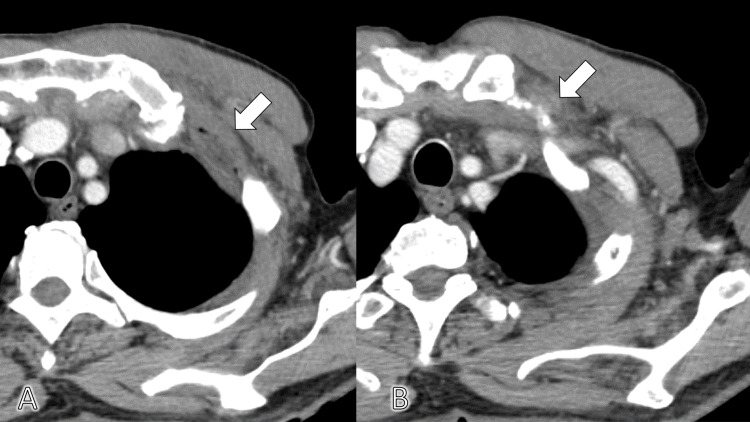
Contrast-enhanced computed tomography Contrast-enhanced computed tomography from the neck to the pelvis showing (A) fluid collection with gas (white arrow) in the first and second intercostal muscles and (B) calcification of the costosternal joint (white arrow).

Contrast-enhanced computed tomography from the neck to the pelvis revealed gas within collections of fluid in the first and second intercostal muscles (Figures 2A, 2B).

**Figure 3:**
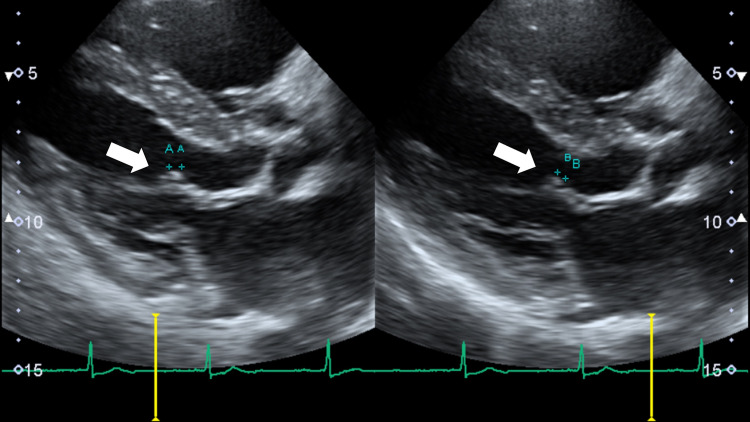
Transthoracic echocardiography on the fourth day of hospitalization revealing a 4–5 mm vegetation on the anterior mitral leaflet (white arrows pointing to the vegetation shown by + +)

We diagnosed multiple abscesses in the left anterior chest muscles and performed aspiration. Gram staining of the pus revealed many leukocytes and Gram-positive cocci, suggesting Staphylococcus and anaerobic bacterial infections. The patient was treated with intravenous ampicillin (ABPC) 6 g, sulbactam (SBT) 9 g, and vancomycin 1 g, covering Methicillin-resistant *S. aureus* and anaerobic bacteria. MSSA was grown from the admission blood culture, so all other antibiotics were stopped, and intravenous cephazolin (CEZ) 4 g was commenced on the third day of hospitalization, following the collection of repeat blood cultures. For the pain in his left chest wall, oral acetaminophen 1,500 mg was started on admission. Transthoracic echocardiography (TTE) on the fourth day of hospitalization revealed a 4-5 mm vegetation on the anterior mitral leaflet (Figure 3).

The diagnosis of IE was made by satisfying two major items of the modified Duke Diagnostic Criteria: two blood cultures that tested positive for *S. aureus* and a valve vegetation seen on TTE. On the fourth day of hospitalization, the patient was referred to the Dental and Oral Surgery Department to rule out an oral source of *S. aureus* infection. He was reported to have good oral hygiene and no dental disease.

**Figure 4:**
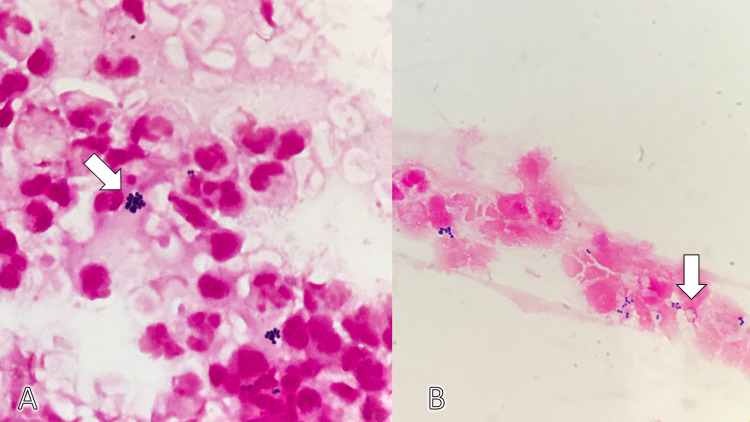
Gram stain Gram stain of pus from the intercostal muscle abscess showing (A) numerous polynuclear leukocytes and Gram-positive cocci (white arrow) and (B) crystals of calcium pyrophosphate (white arrow).

On the fifth day of hospitalization, the patient developed swelling of the left anterior chest wall, low-grade fever, and his left-sided chest pain continued despite taking regular doses of acetaminophen. The abscess in the left second intercostal muscle was drained, and a small amount of cream-colored pus was aspirated. We suspected the involvement of anaerobic bacteria and changed the antibacterial drugs from CEZ back to ABPC/SBT. We found numerous polynuclear leukocytes and Gram-positive cocci scattered in the pus Gram stain, as well as crystals of calcium pyrophosphate (Figures 4A, 4B).

Considering the possibility of intramuscular pseudogout, pain control was changed from acetaminophen to diclofenac from the seventh day of hospitalization. After this change in treatment, the patient’s pain improved, and his fever resolved. Repeat blood cultures on days 4 and 9 were tested negative. When a TTE was repeated on day 11, the valve vegetation on the anterior mitral leaflet had disappeared, and no evidence of valve destruction was observed. On day 14, diclofenac administration was stopped because the pain in the left anterior chest had dissipated. As there was no valve destruction and the patient’s symptoms were stable, the ABPC/SBT infusion was stopped after two weeks and switched to oral trimethoprim-sulfamethoxazole on discharge from the hospital on the 15th day of hospitalization. He was followed up in the outpatient department four weeks later, with no reoccurrence of IE or intercostal abscesses.

## Discussion

This case shows that IE can occur with intercostal muscle abscesses triggered by the deposition of calcium pyrophosphate crystals. Abscess formation in muscles can be triggered by calcium pyrophosphate deposition. In general, most intercostal muscle abscesses are caused by direct invasion [11,14]. However, in this case, no lung lesions were observed, and IE was suspected because *S. aureus* was cultured from the intercostal muscle abscess and the blood. In addition, as calcium pyrophosphate was present simultaneously, nonsteroidal anti-inflammatory drugs (NSAIDs) and drainage and antibacterial agents were effective treatments.

In this case, there were concurrent intercostal muscle abscesses seeded from the bloodstream and IE. Calcium pyrophosphate may enhance susceptibility to infection and make treatment difficult [13]. Treatment of pseudogout may be effective in the treatment of abscesses associated with calcium pyrophosphate [13].

For a diagnosis of IE, identification of the bacterial entry point is important in terms of infection control. There is a high possibility of the infective source being the oral cavity or skin [2]. In the present case, there was no skin damage around the intercostal muscles and no lung lesions; therefore, it was unlikely that the intercostal muscle was an entry point. There were no abnormalities with the oral mucosa and neck, and as there was no swelling of the lymph nodes, the possibility of infection from the oral cavity was low. However, as there was a history of a burn seven weeks before this admission, the bloodstream infection could have been caused by *S. aureus*, which is part of the skin microbiota, from the burn site, followed by IE [3].

The standard treatment for IE due to MSSA bacteremia is CEZ, depending on the clinical course, as in this case [2,7]. CEZ was selected based on the antibiotic sensitivity results of the blood and abscess cultures. However, because of the exacerbation of the symptoms, the patient was recommenced on ABPC/SBT antibiotics for four weeks, and repeat echocardiography was performed. As calcium pyrophosphate was observed microscopically in the pus aspirated from the intercostal muscle abscesses, it is possible that inflammation was also induced by the intramuscular accumulation of calcium pyrophosphate [15,16]. NSAIDs may have contributed to a reduction in pain and swelling.

Accumulation of calcium pyrophosphate in muscles can cause abscess formation. In this case, the origin of the intercostal muscle abscess was near the sternoclavicular and sternocostal joints, and calcium pyrophosphate could have been deposited in the muscle at those sites [17]. Previous studies have shown that calcium pyrophosphate deposition can trigger inflammation and abscess formation [13,16,18,19]. Calcium pyrophosphate has been observed in an iliopsoas abscess associated with intervertebral discitis, suggesting the possibility of migratory pseudogout [13]. As far as we are aware, there are no previous reports on calcium pyrophosphate deposition in intercostal muscle abscesses. As the relationship between the deposition of calcium pyrophosphate and infection has not been clearly investigated, it is necessary to study future cases.

## Conclusions

IE can form an abscess in the intercostal muscles. Careful follow-up is required in patients with IE, due to the possibility for abscess formation. Furthermore, calcium pyrophosphate deposition in muscles around joints can trigger abscess formation when bloodstream infections are present.
